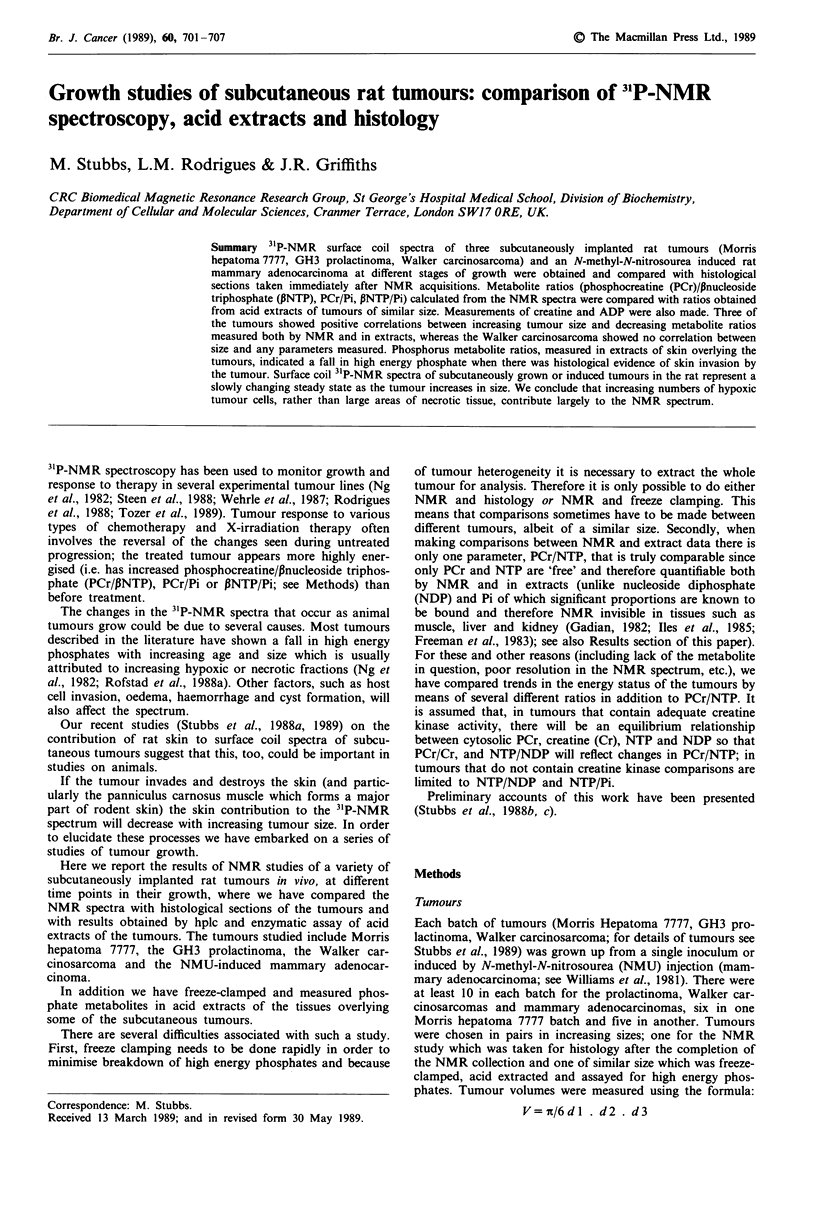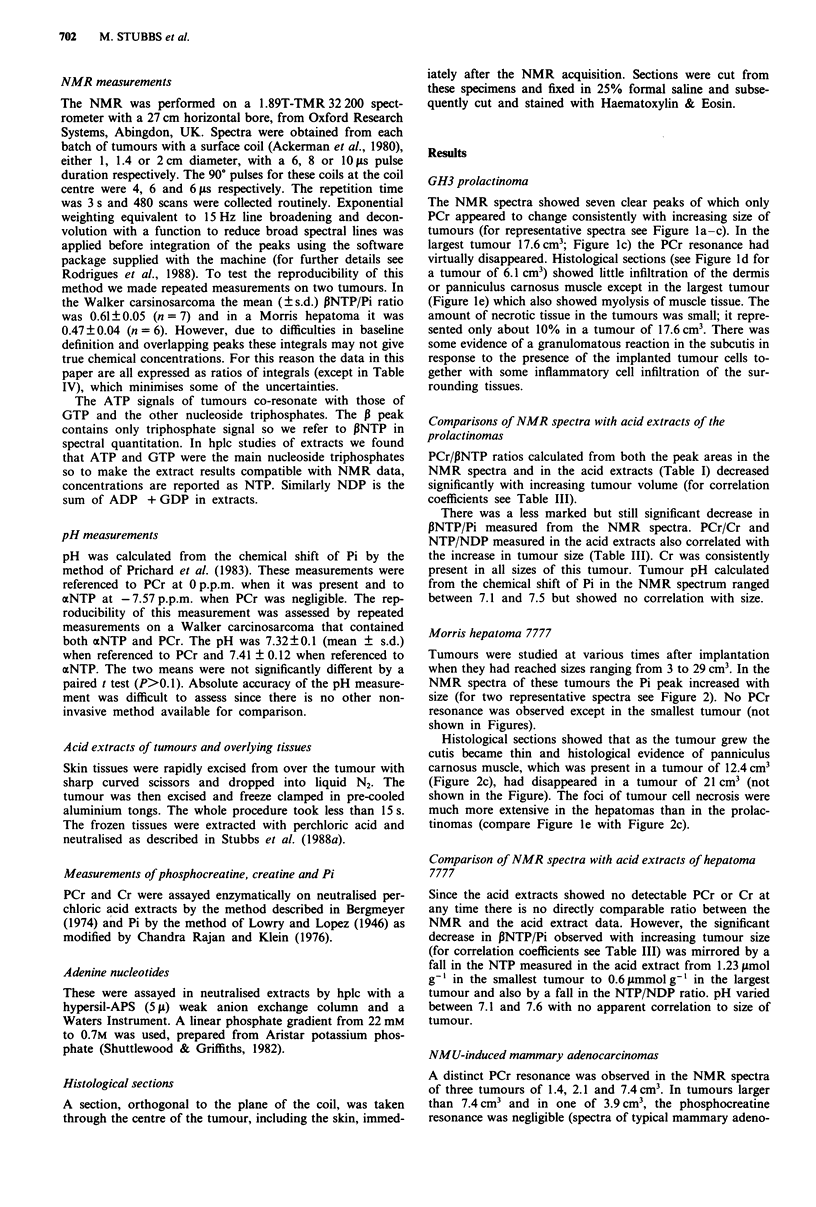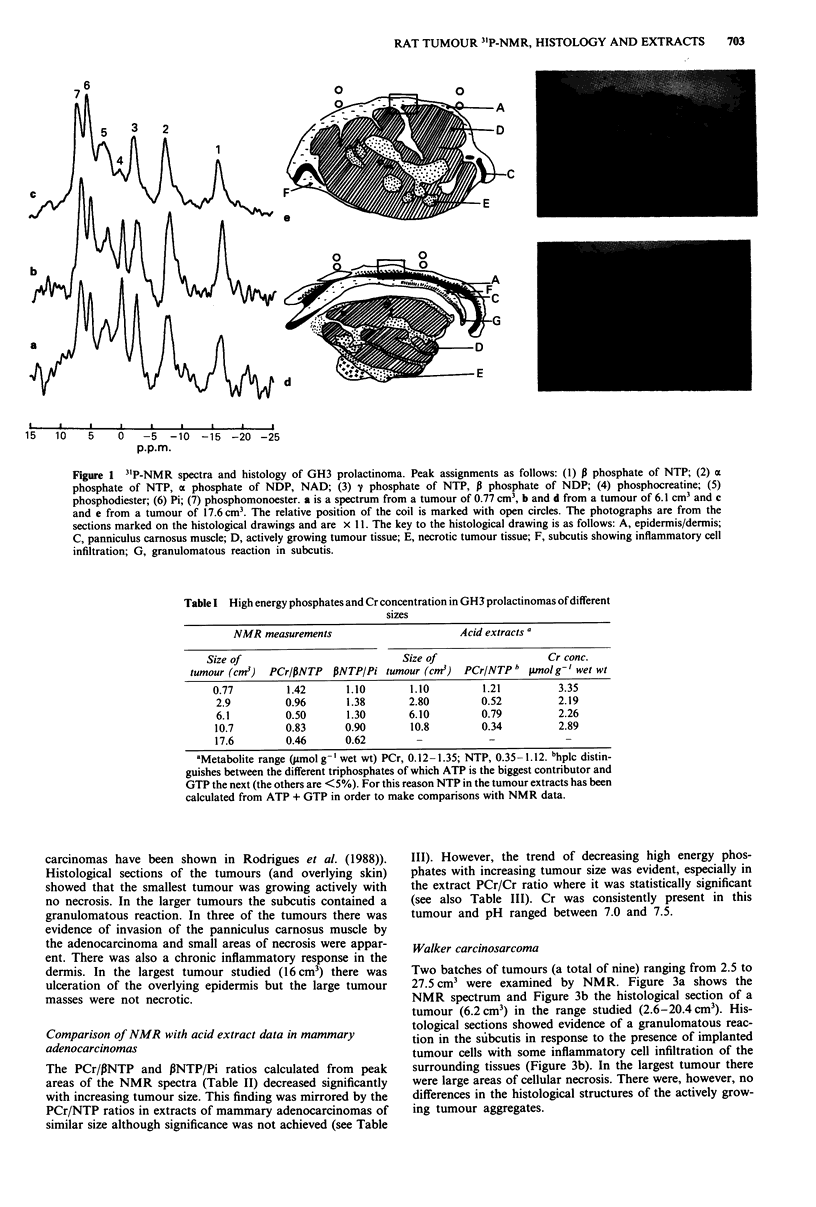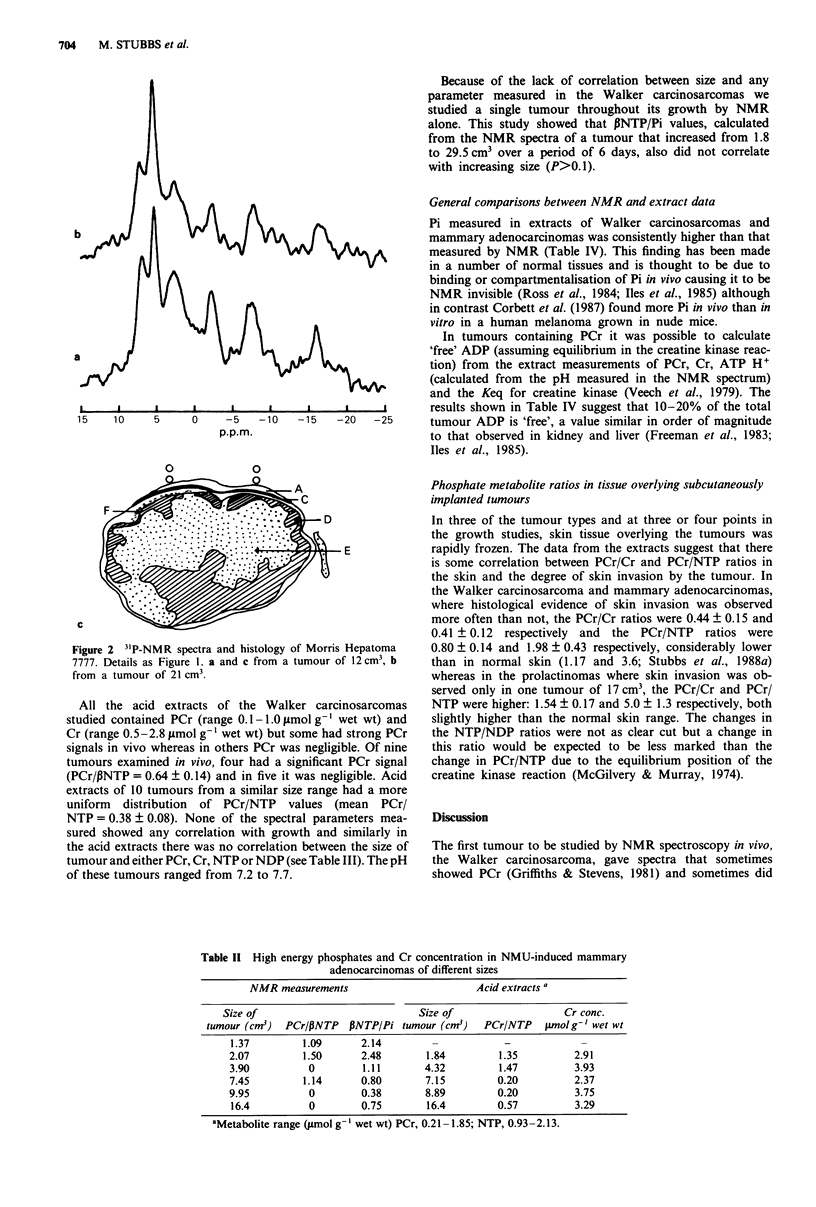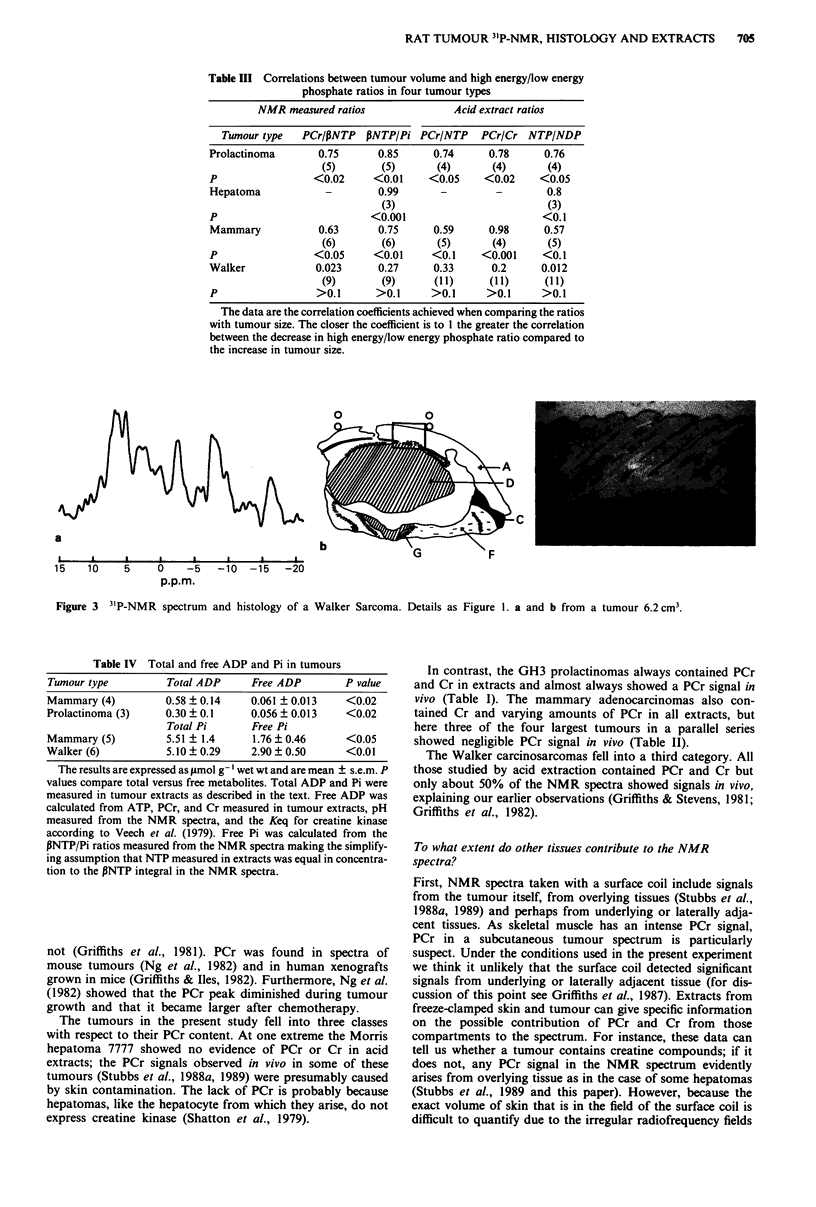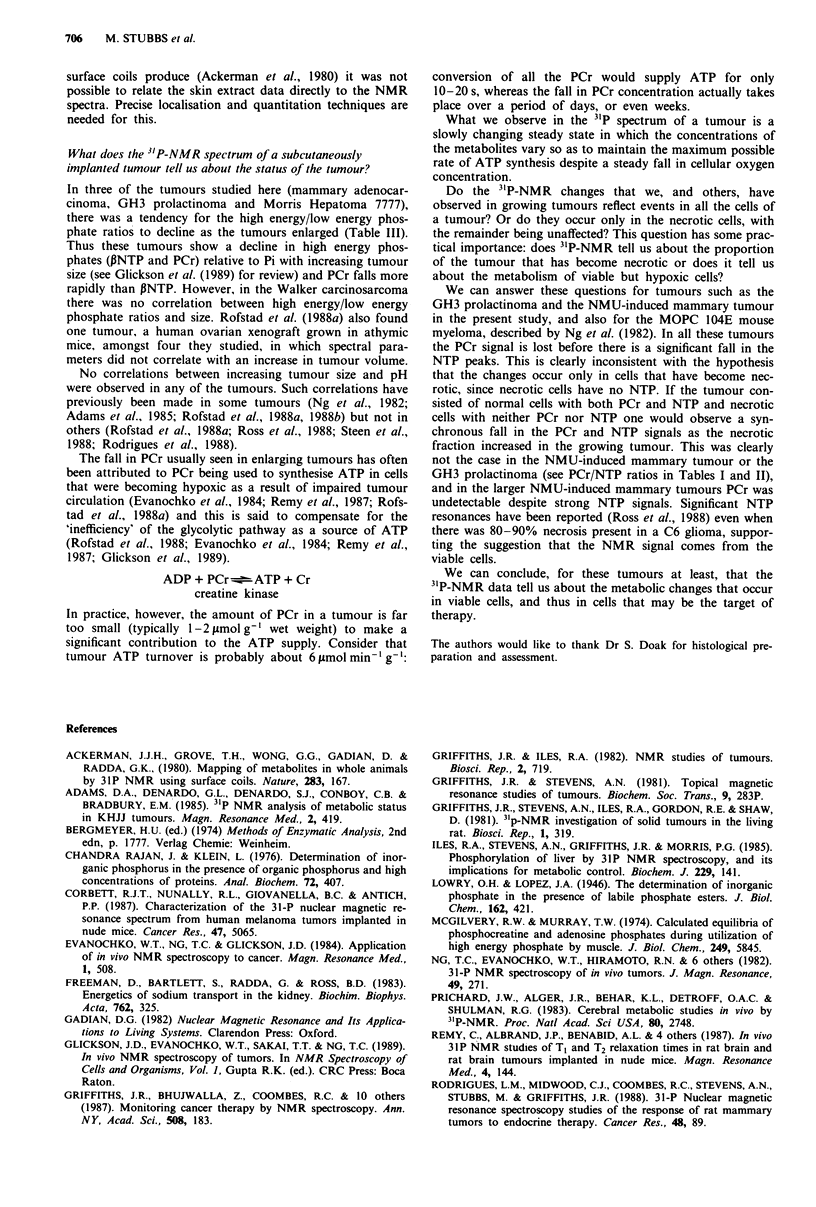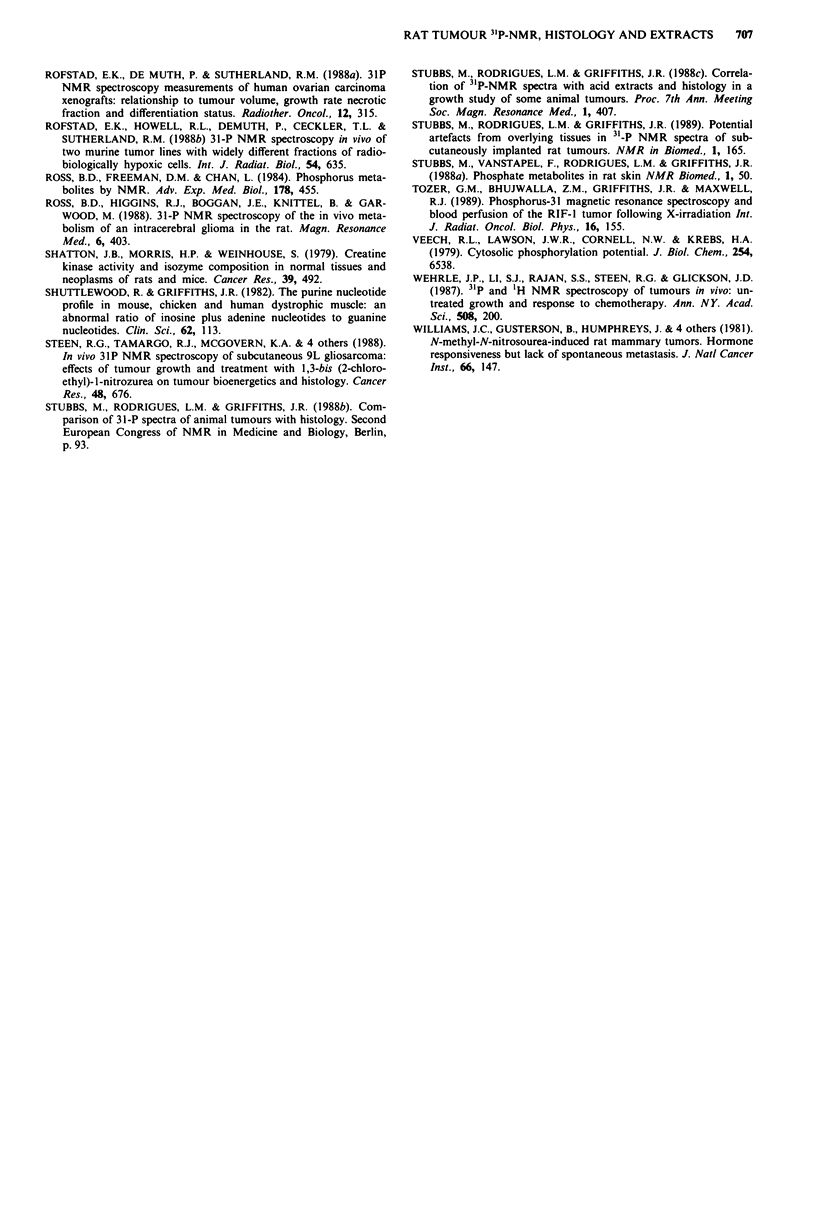# Growth studies of subcutaneous rat tumours: comparison of 31P-NMR spectroscopy, acid extracts and histology.

**DOI:** 10.1038/bjc.1989.343

**Published:** 1989-11

**Authors:** M. Stubbs, L. M. Rodrigues, J. R. Griffiths

**Affiliations:** CRC Biomedical Magnetic Resonance Research Group, St George's Hospital Medical School, London, UK.

## Abstract

**Images:**


Br. J. Cancer (1989), 60, 701-707 © The Macmillan Press Ltd., 1989

### Growth studies of subcutaneous rat tumours: comparison of $^{31}$P-NMR spectroscopy, acid extracts and histology

M. Stubbs, L.M. Rodrigues & J.R. Griffiths

*CRC Biomedical Magnetic Resonance Research Group, St George's Hospital Medical School, Division of Biochemistry, Department of Cellular and Molecular Sciences, Cranmer Terrace, London SW17 0RE, UK.*

**Summary** $^{31}$P-NMR surface coil spectra of three subcutaneously implanted rat tumours (Morris hepatoma 7777, GH3 prolactinoma, Walker carcinosarcoma) and an *N*-methyl-*N*-nitrosourea induced rat mammary adenocarcinoma at different stages of growth were obtained and compared with histological sections taken immediately after NMR acquisitions. Metabolite ratios (phosphocreatine (PCr)/βnucleoside triphosphate (βNTP), PCr/Pi, βNTP/Pi) calculated from the NMR spectra were compared with ratios obtained from acid extracts of tumours of similar size. Measurements of creatine and ADP were also made. Three of the tumours showed positive correlations between increasing tumour size and decreasing metabolite ratios measured both by NMR and in extracts, whereas the Walker carcinosarcoma showed no correlation between size and any parameters measured. Phosphorus metabolite ratios, measured in extracts of skin overlying the tumours, indicated a fall in high energy phosphate when there was histological evidence of skin invasion by the tumour. Surface coil $^{31}$P-NMR spectra of subcutaneously grown or induced tumours in the rat represent a slowly changing steady state as the tumour increases in size. We conclude that increasing numbers of hypoxic tumour cells, rather than large areas of necrotic tissue, contribute largely to the NMR spectrum.

$^{31}$P-NMR spectroscopy has been used to monitor growth and response to therapy in several experimental tumour lines (Ng *et al.*, 1982; Steen *et al.*, 1988; Wehrle *et al.*, 1987; Rodrigues *et al.*, 1988; Tozer *et al.*, 1989). Tumour response to various types of chemotherapy and X-irradiation therapy often involves the reversal of the changes seen during untreated progression; the treated tumour appears more highly energised (i.e. has increased phosphocreatine/βnucleoside triphosphate (PCr/βNTP), PCr/Pi or βNTP/Pi; see Methods) than before treatment.

The changes in the $^{31}$P-NMR spectra that occur as animal tumours grow could be due to several causes. Most tumours described in the literature have shown a fall in high energy phosphates with increasing age and size which is usually attributed to increasing hypoxic or necrotic fractions (Ng *et al.*, 1982; Rofstad *et al.*, 1988a). Other factors, such as host cell invasion, oedema, haemorrhage and cyst formation, will also affect the spectrum.

Our recent studies (Stubbs *et al.*, 1988*a*, 1989) on the contribution of rat skin to surface coil spectra of subcutaneous tumours suggest that this, too, could be important in studies on animals.

If the tumour invades and destroys the skin (and particularly the panniculus carnosus muscle which forms a major part of rodent skin) the skin contribution to the $^{31}$P-NMR spectrum will decrease with increasing tumour size. In order to elucidate these processes we have embarked on a series of studies of tumour growth.

Here we report the results of NMR studies of a variety of subcutaneously implanted rat tumours *in vivo*, at different time points in their growth, where we have compared the NMR spectra with histological sections of the tumours and with results obtained by hplc and enzymatic assay of acid extracts of the tumours. The tumours studied include Morris hepatoma 7777, the GH3 prolactinoma, the Walker carcinosarcoma and the NMU-induced mammary adenocarcinoma.

In addition we have freeze-clamped and measured phosphate metabolites in acid extracts of the tissues overlying some of the subcutaneous tumours.

There are several difficulties associated with such a study. First, freeze clamping needs to be done rapidly in order to minimise breakdown of high energy phosphates and because of tumour heterogeneity it is necessary to extract the whole tumour for analysis. Therefore it is only possible to do either NMR and histology *or* NMR and freeze clamping. This means that comparisons sometimes have to be made between different tumours, albeit of a similar size. Secondly, when making comparisons between NMR and extract data there is only one parameter, PCr/NTP, that is truly comparable since only PCr and NTP are 'free' and therefore quantifiable both by NMR and in extracts (unlike nucleoside diphosphate (NDP) and Pi of which significant proportions are known to be bound and therefore NMR invisible in tissues such as muscle, liver and kidney (Gadian, 1982; Iles *et al.*, 1985; Freeman *et al.*, 1983); see also Results section of this paper). For these and other reasons (including lack of the metabolite in question, poor resolution in the NMR spectrum, etc.), we have compared trends in the energy status of the tumours by means of several different ratios in addition to PCr/NTP. It is assumed that, in tumours that contain adequate creatine kinase activity, there will be an equilibrium relationship between cytosolic PCr, creatine (Cr), NTP and NDP so that PCr/Cr, and NTP/NDP will reflect changes in PCr/NTP; in tumours that do not contain creatine kinase comparisons are limited to NTP/NDP and NTP/Pi.

Preliminary accounts of this work have been presented (Stubbs *et al.*, 1988*b*, *c*).

### Methods

### *Tumours*

Each batch of tumours (Morris Hepatoma 7777, GH3 prolactinoma, Walker carcinosarcoma; for details of tumours see Stubbs *et al.*, 1989) was grown up from a single inoculum or induced by *N*-methyl-*N*-nitrosourea (NMU) injection (mammary adenocarcinoma; see Williams *et al.*, 1981). There were at least 10 in each batch for the prolactinoma, Walker carcinosarcomas and mammary adenocarcinomas, six in one Morris hepatoma 7777 batch and five in another. Tumours were chosen in pairs in increasing sizes; one for the NMR study which was taken for histology after the completion of the NMR collection and one of similar size which was freeze-clamped, acid extracted and assayed for high energy phosphates. Tumour volumes were measured using the formula:

$$V = \pi/6\, d1 \,.\, d2 \,.\, d3$$

Correspondence: M. Stubbs.
Received 13 March 1989; and in revised form 30 May 1989.

*NMR measurements*

The NMR was performed on a 1.89T-TMR 32 200 spectrometer with a 27 cm horizontal bore, from Oxford Research Systems, Abingdon, UK. Spectra were obtained from each batch of tumours with a surface coil (Ackerman *et al.*, 1980), either 1, 1.4 or 2 cm diameter, with a 6, 8 or 10 μs pulse duration respectively. The 90° pulses for these coils at the coil centre were 4, 6 and 6 μs respectively. The repetition time was 3 s and 480 scans were collected routinely. Exponential weighting equivalent to 15 Hz line broadening and deconvolution with a function to reduce broad spectral lines was applied before integration of the peaks using the software package supplied with the machine (for further details see Rodrigues *et al.*, 1988). To test the reproducibility of this method we made repeated measurements on two tumours. In the Walker carsinosarcoma the mean (± s.d.) βNTP/Pi ratio was 0.61 ± 0.05 ($n = 7$) and in a Morris hepatoma it was 0.47 ± 0.04 ($n = 6$). However, due to difficulties in baseline definition and overlapping peaks these integrals may not give true chemical concentrations. For this reason the data in this paper are all expressed as ratios of integrals (except in Table IV), which minimises some of the uncertainties.

The ATP signals of tumours co-resonate with those of GTP and the other nucleoside triphosphates. The β peak contains only triphosphate signal so we refer to βNTP in spectral quantitation. In hplc studies of extracts we found that ATP and GTP were the main nucleoside triphosphates so to make the extract results compatible with NMR data, concentrations are reported as NTP. Similarly NDP is the sum of ADP + GDP in extracts.

*pH measurements*

pH was calculated from the chemical shift of Pi by the method of Prichard *et al.* (1983). These measurements were referenced to PCr at 0 p.p.m. when it was present and to αNTP at −7.57 p.p.m. when PCr was negligible. The reproducibility of this measurement was assessed by repeated measurements on a Walker carcinosarcoma that contained both αNTP and PCr. The pH was 7.32 ± 0.1 (mean ± s.d.) when referenced to PCr and 7.41 ± 0.12 when referenced to αNTP. The two means were not significantly different by a paired *t* test ($P > 0.1$). Absolute accuracy of the pH measurement was difficult to assess since there is no other non-invasive method available for comparison.

*Acid extracts of tumours and overlying tissues*

Skin tissues were rapidly excised from over the tumour with sharp curved scissors and dropped into liquid $N_2$. The tumour was then excised and freeze clamped in pre-cooled aluminium tongs. The whole procedure took less than 15 s. The frozen tissues were extracted with perchloric acid and neutralised as described in Stubbs *et al.* (1988*a*).

*Measurements of phosphocreatine, creatine and Pi*

PCr and Cr were assayed enzymatically on neutralised perchloric acid extracts by the method described in Bergmeyer (1974) and Pi by the method of Lowry and Lopez (1946) as modified by Chandra Rajan and Klein (1976).

*Adenine nucleotides*

These were assayed in neutralised extracts by hplc with a hypersil-APS (5 μ) weak anion exchange column and a Waters Instrument. A linear phosphate gradient from 22 mM to 0.7M was used, prepared from Aristar potassium phosphate (Shuttlewood & Griffiths, 1982).

*Histological sections*

A section, orthogonal to the plane of the coil, was taken through the centre of the tumour, including the skin, immediately after the NMR acquisition. Sections were cut from these specimens and fixed in 25% formal saline and subsequently cut and stained with Haematoxylin & Eosin.

### Results

*GH3 prolactinoma*

The NMR spectra showed seven clear peaks of which only PCr appeared to change consistently with increasing size of tumours (for representative spectra see Figure 1a–c). In the largest tumour 17.6 $cm^3$; Figure 1c) the PCr resonance had virtually disappeared. Histological sections (see Figure 1d for a tumour of 6.1 $cm^3$) showed little infiltration of the dermis or panniculus carnosus muscle except in the largest tumour (Figure 1e) which also showed myolysis of muscle tissue. The amount of necrotic tissue in the tumours was small; it represented only about 10% in a tumour of 17.6 $cm^3$. There was some evidence of a granulomatous reaction in the subcutis in response to the presence of the implanted tumour cells together with some inflammatory cell infiltration of the surrounding tissues.

*Comparisons of NMR spectra with acid extracts of the prolactinomas*

PCr/βNTP ratios calculated from both the peak areas in the NMR spectra and in the acid extracts (Table I) decreased significantly with increasing tumour volume (for correlation coefficients see Table III).

There was a less marked but still significant decrease in βNTP/Pi measured from the NMR spectra. PCr/Cr and NTP/NDP measured in the acid extracts also correlated with the increase in tumour size (Table III). Cr was consistently present in all sizes of this tumour. Tumour pH calculated from the chemical shift of Pi in the NMR spectrum ranged between 7.1 and 7.5 but showed no correlation with size.

*Morris hepatoma 7777*

Tumours were studied at various times after implantation when they had reached sizes ranging from 3 to 29 $cm^3$. In the NMR spectra of these tumours the Pi peak increased with size (for two representative spectra see Figure 2). No PCr resonance was observed except in the smallest tumour (not shown in Figures).

Histological sections showed that as the tumour grew the cutis became thin and histological evidence of panniculus carnosus muscle, which was present in a tumour of 12.4 $cm^3$ (Figure 2c), had disappeared in a tumour of 21 $cm^3$ (not shown in the Figure). The foci of tumour cell necrosis were much more extensive in the hepatomas than in the prolactinomas (compare Figure 1e with Figure 2c).

*Comparison of NMR spectra with acid extracts of hepatoma 7777*

Since the acid extracts showed no detectable PCr or Cr at any time there is no directly comparable ratio between the NMR and the acid extract data. However, the significant decrease in βNTP/Pi observed with increasing tumour size (for correlation coefficients see Table III) was mirrored by a fall in the NTP measured in the acid extract from 1.23 μmol $g^{-1}$ in the smallest tumour to 0.6 μmmol $g^{-1}$ in the largest tumour and also by a fall in the NTP/NDP ratio. pH varied between 7.1 and 7.6 with no apparent correlation to size of tumour.

*NMU-induced mammary adenocarcinomas*

A distinct PCr resonance was observed in the NMR spectra of three tumours of 1.4, 2.1 and 7.4 $cm^3$. In tumours larger than 7.4 $cm^3$ and in one of 3.9 $cm^3$, the phosphocreatine resonance was negligible (spectra of typical mammary adeno-

**Figure 1** $^{31}$P-NMR spectra and histology of GH3 prolactinoma. Peak assignments as follows: (1) $\beta$ phosphate of NTP; (2) $\alpha$ phosphate of NTP, $\alpha$ phosphate of NDP, NAD; (3) $\gamma$ phosphate of NTP, $\beta$ phosphate of NDP; (4) phosphocreatine; (5) phosphodiester; (6) Pi; (7) phosphomonoester. **a** is a spectrum from a tumour of 0.77 $cm^3$, **b** and **d** from a tumour of 6.1 $cm^3$ and **c** and **e** from a tumour of 17.6 $cm^3$. The relative position of the coil is marked with open circles. The photographs are from the sections marked on the histological drawings and are × 11. The key to the histological drawing is as follows: A, epidermis/dermis; C, panniculus carnosus muscle; D, actively growing tumour tissue; E, necrotic tumour tissue; F, subcutis showing inflammatory cell infiltration; G, granulomatous reaction in subcutis.

**Table I** High energy phosphates and Cr concentration in GH3 prolactinomas of different sizes

| *NMR measurements* | | | *Acid extracts* [a] | | |
|---|---|---|---|---|---|
| *Size of tumour ($cm^3$)* | *PCr/βNTP* | *βNTP/Pi* | *Size of tumour ($cm^3$)* | *PCr/NTP* [b] | *Cr conc. μmol $g^{-1}$ wet wt* |
| 0.77 | 1.42 | 1.10 | 1.10 | 1.21 | 3.35 |
| 2.9 | 0.96 | 1.38 | 2.80 | 0.52 | 2.19 |
| 6.1 | 0.50 | 1.30 | 6.10 | 0.79 | 2.26 |
| 10.7 | 0.83 | 0.90 | 10.8 | 0.34 | 2.89 |
| 17.6 | 0.46 | 0.62 | – | – | – |

[a]Metabolite range (μmol $g^{-1}$ wet wt) PCr, 0.12–1.35; NTP, 0.35–1.12. [b]hplc distinguishes between the different triphosphates of which ATP is the biggest contributor and GTP the next (the others are <5%). For this reason NTP in the tumour extracts has been calculated from ATP + GTP in order to make comparisons with NMR data.

carcinomas have been shown in Rodrigues *et al.* (1988)). Histological sections of the tumours (and overlying skin) showed that the smallest tumour was growing actively with no necrosis. In the larger tumours the subcutis contained a granulomatous reaction. In three of the tumours there was evidence of invasion of the panniculus carnosus muscle by the adenocarcinoma and small areas of necrosis were apparent. There was also a chronic inflammatory response in the dermis. In the largest tumour studied (16 $cm^3$) there was ulceration of the overlying epidermis but the large tumour masses were not necrotic.

### *Comparison of NMR with acid extract data in mammary adenocarcinomas*

The PCr/βNTP and βNTP/Pi ratios calculated from peak areas of the NMR spectra (Table II) decreased significantly with increasing tumour size. This finding was mirrored by the PCr/NTP ratios in extracts of mammary adenocarcinomas of similar size although significance was not achieved (see Table III). However, the trend of decreasing high energy phosphates with increasing tumour size was evident, especially in the extract PCr/Cr ratio where it was statistically significant (see also Table III). Cr was consistently present in this tumour and pH ranged between 7.0 and 7.5.

### *Walker carcinosarcoma*

Two batches of tumours (a total of nine) ranging from 2.5 to 27.5 $cm^3$ were examined by NMR. Figure 3a shows the NMR spectrum and Figure 3b the histological section of a tumour (6.2 $cm^3$) in the range studied (2.6–20.4 $cm^3$). Histological sections showed evidence of a granulomatous reaction in the subcutis in response to the presence of implanted tumour cells with some inflammatory cell infiltration of the surrounding tissues (Figure 3b). In the largest tumour there were large areas of cellular necrosis. There were, however, no differences in the histological structures of the actively growing tumour aggregates.

**Figure 2** $^{31}$P-NMR spectra and histology of Morris Hepatoma 7777. Details as Figure 1. **a** and **c** from a tumour of 12 cm$^3$, **b** from a tumour of 21 cm$^3$.

All the acid extracts of the Walker carcinosarcomas studied contained PCr (range 0.1–1.0 μmol g$^{-1}$ wet wt) and Cr (range 0.5–2.8 μmol g$^{-1}$ wet wt) but some had strong PCr signals in vivo whereas in others PCr was negligible. Of nine tumours examined *in vivo*, four had a significant PCr signal (PCr/βNTP = 0.64 ± 0.14) and in five it was negligible. Acid extracts of 10 tumours from a similar size range had a more uniform distribution of PCr/NTP values (mean PCr/NTP = 0.38 ± 0.08). None of the spectral parameters measured showed any correlation with growth and similarly in the acid extracts there was no correlation between the size of tumour and either PCr, Cr, NTP or NDP (see Table III). The pH of these tumours ranged from 7.2 to 7.7.

Because of the lack of correlation between size and any parameter measured in the Walker carcinosarcomas we studied a single tumour throughout its growth by NMR alone. This study showed that βNTP/Pi values, calculated from the NMR spectra of a tumour that increased from 1.8 to 29.5 cm$^3$ over a period of 6 days, also did not correlate with increasing size ($P$>0.1).

### *General comparisons between NMR and extract data*

Pi measured in extracts of Walker carcinosarcomas and mammary adenocarcinomas was consistently higher than that measured by NMR (Table IV). This finding has been made in a number of normal tissues and is thought to be due to binding or compartmentalisation of Pi *in vivo* causing it to be NMR invisible (Ross *et al.*, 1984; Iles *et al.*, 1985) although in contrast Corbett *et al.* (1987) found more Pi *in vivo* than *in vitro* in a human melanoma grown in nude mice.

In tumours containing PCr it was possible to calculate 'free' ADP (assuming equilibrium in the creatine kinase reaction) from the extract measurements of PCr, Cr, ATP $H^+$ (calculated from the pH measured in the NMR spectrum) and the *K*eq for creatine kinase (Veech *et al.*, 1979). The results shown in Table IV suggest that 10–20% of the total tumour ADP is 'free', a value similar in order of magnitude to that observed in kidney and liver (Freeman *et al.*, 1983; Iles *et al.*, 1985).

### *Phosphate metabolite ratios in tissue overlying subcutaneously implanted tumours*

In three of the tumour types and at three or four points in the growth studies, skin tissue overlying the tumours was rapidly frozen. The data from the extracts suggest that there is some correlation between PCr/Cr and PCr/NTP ratios in the skin and the degree of skin invasion by the tumour. In the Walker carcinosarcoma and mammary adenocarcinomas, where histological evidence of skin invasion was observed more often than not, the PCr/Cr ratios were 0.44 ± 0.15 and 0.41 ± 0.12 respectively and the PCr/NTP ratios were 0.80 ± 0.14 and 1.98 ± 0.43 respectively, considerably lower than in normal skin (1.17 and 3.6; Stubbs *et al.*, 1988*a*) whereas in the prolactinomas where skin invasion was observed only in one tumour of 17 cm$^3$, the PCr/Cr and PCr/NTP were higher: 1.54 ± 0.17 and 5.0 ± 1.3 respectively, both slightly higher than the normal skin range. The changes in the NTP/NDP ratios were not as clear cut but a change in this ratio would be expected to be less marked than the change in PCr/NTP due to the equilibrium position of the creatine kinase reaction (McGilvery & Murray, 1974).

### Discussion

The first tumour to be studied by NMR spectroscopy *in vivo*, the Walker carcinosarcoma, gave spectra that sometimes showed PCr (Griffiths & Stevens, 1981) and sometimes did

**Table II** High energy phosphates and Cr concentration in NMU-induced mammary adenocarcinomas of different sizes

| *NMR measurements* | | | *Acid extracts* [a] | | |
|---|---|---|---|---|---|
| *Size of tumour (cm³)* | *PCr/βNTP* | *βNTP/Pi* | *Size of tumour (cm³)* | *PCr/NTP* | *Cr conc. μmol g⁻¹ wet wt* |
| 1.37 | 1.09 | 2.14 | – | – | – |
| 2.07 | 1.50 | 2.48 | 1.84 | 1.35 | 2.91 |
| 3.90 | 0 | 1.11 | 4.32 | 1.47 | 3.93 |
| 7.45 | 1.14 | 0.80 | 7.15 | 0.20 | 2.37 |
| 9.95 | 0 | 0.38 | 8.89 | 0.20 | 3.75 |
| 16.4 | 0 | 0.75 | 16.4 | 0.57 | 3.29 |

[a]Metabolite range (μmol g$^{-1}$ wet wt) PCr, 0.21–1.85; NTP, 0.93–2.13.

**Table III** Correlations between tumour volume and high energy/low energy phosphate ratios in four tumour types

| | *NMR measured ratios* | | *Acid extract ratios* | | |
|---|---|---|---|---|---|
| *Tumour type* | *PCr/βNTP* | *βNTP/Pi* | *PCr/NTP* | *PCr/Cr* | *NTP/NDP* |
| Prolactinoma | 0.75 | 0.85 | 0.74 | 0.78 | 0.76 |
| | (5) | (5) | (4) | (4) | (4) |
| *P* | <0.02 | <0.01 | <0.05 | <0.02 | <0.05 |
| Hepatoma | – | 0.99 | – | – | 0.8 |
| | | (3) | | | (3) |
| *P* | | <0.001 | | | <0.1 |
| Mammary | 0.63 | 0.75 | 0.59 | 0.98 | 0.57 |
| | (6) | (6) | (5) | (4) | (5) |
| *P* | <0.05 | <0.01 | <0.1 | <0.001 | <0.1 |
| Walker | 0.023 | 0.27 | 0.33 | 0.2 | 0.012 |
| | (9) | (9) | (11) | (11) | (11) |
| *P* | >0.1 | >0.1 | >0.1 | >0.1 | >0.1 |

The data are the correlation coefficients achieved when comparing the ratios with tumour size. The closer the coefficient is to 1 the greater the correlation between the decrease in high energy/low energy phosphate ratio compared to the increase in tumour size.

**Figure 3** ³¹P-NMR spectrum and histology of a Walker Sarcoma. Details as Figure 1. **a** and **b** from a tumour 6.2 $cm^3$.

**Table IV** Total and free ADP and Pi in tumours

| *Tumour type* | *Total ADP* | *Free ADP* | *P value* |
|---|---|---|---|
| Mammary (4) | 0.58 ± 0.14 | 0.061 ± 0.013 | <0.02 |
| Prolactinoma (3) | 0.30 ± 0.1 | 0.056 ± 0.013 | <0.02 |
| | *Total Pi* | *Free Pi* | |
| Mammary (5) | 5.51 ± 1.4 | 1.76 ± 0.46 | <0.05 |
| Walker (6) | 5.10 ± 0.29 | 2.90 ± 0.50 | <0.01 |

The results are expressed as μmol $g^{-1}$ wet wt and are mean ± s.e.m. *P* values compare total versus free metabolites. Total ADP and Pi were measured in tumour extracts as described in the text. Free ADP was calculated from ATP, PCr, and Cr measured in tumour extracts, pH measured from the NMR spectra, and the *K*eq for creatine kinase according to Veech *et al.* (1979). Free Pi was calculated from the βNTP/Pi ratios measured from the NMR spectra making the simplifying assumption that NTP measured in extracts was equal in concentration to the βNTP integral in the NMR spectra.

not (Griffiths *et al.*, 1981). PCr was found in spectra of mouse tumours (Ng *et al.*, 1982) and in human xenografts grown in mice (Griffiths & Iles, 1982). Furthermore, Ng *et al.* (1982) showed that the PCr peak diminished during tumour growth and that it became larger after chemotherapy.

The tumours in the present study fell into three classes with respect to their PCr content. At one extreme the Morris hepatoma 7777 showed no evidence of PCr or Cr in acid extracts; the PCr signals observed *in vivo* in some of these tumours (Stubbs *et al.*, 1988*a*, 1989) were presumably caused by skin contamination. The lack of PCr is probably because hepatomas, like the hepatocyte from which they arise, do not express creatine kinase (Shatton *et al.*, 1979).

In contrast, the GH3 prolactinomas always contained PCr and Cr in extracts and almost always showed a PCr signal *in vivo* (Table I). The mammary adenocarcinomas also contained Cr and varying amounts of PCr in all extracts, but here three of the four largest tumours in a parallel series showed negligible PCr signal *in vivo* (Table II).

The Walker carcinosarcomas fell into a third category. All those studied by acid extraction contained PCr and Cr but only about 50% of the NMR spectra showed signals *in vivo*, explaining our earlier observations (Griffiths & Stevens, 1981; Griffiths *et al.*, 1982).

### *To what extent do other tissues contribute to the NMR spectra?*

First, NMR spectra taken with a surface coil include signals from the tumour itself, from overlying tissues (Stubbs *et al.*, 1988*a*, 1989) and perhaps from underlying or laterally adjacent tissues. As skeletal muscle has an intense PCr signal, PCr in a subcutaneous tumour spectrum is particularly suspect. Under the conditions used in the present experiment we think it unlikely that the surface coil detected significant signals from underlying or laterally adjacent tissue (for discussion of this point see Griffiths *et al.*, 1987). Extracts from freeze-clamped skin and tumour can give specific information on the possible contribution of PCr and Cr from those compartments to the spectrum. For instance, these data can tell us whether a tumour contains creatine compounds; if it does not, any PCr signal in the NMR spectrum evidently arises from overlying tissue as in the case of some hepatomas (Stubbs *et al.*, 1989 and this paper). However, because the exact volume of skin that is in the field of the surface coil is difficult to quantify due to the irregular radiofrequency fields surface coils produce (Ackerman et al., 1980) it was not possible to relate the skin extract data directly to the NMR spectra. Precise localisation and quantitation techniques are needed for this.

*What does the $^{31}$P-NMR spectrum of a subcutaneously implanted tumour tell us about the status of the tumour?*

In three of the tumours studied here (mammary adenocarcinoma, GH3 prolactinoma and Morris Hepatoma 7777), there was a tendency for the high energy/low energy phosphate ratios to decline as the tumours enlarged (Table III). Thus these tumours show a decline in high energy phosphates (βNTP and PCr) relative to Pi with increasing tumour size (see Glickson et al. (1989) for review) and PCr falls more rapidly than βNTP. However, in the Walker carcinosarcoma there was no correlation between high energy/low energy phosphate ratios and size. Rofstad et al. (1988*a*) also found one tumour, a human ovarian xenograft grown in athymic mice, amongst four they studied, in which spectral parameters did not correlate with an increase in tumour volume.

No correlations between increasing tumour size and pH were observed in any of the tumours. Such correlations have previously been made in some tumours (Ng et al., 1982; Adams et al., 1985; Rofstad et al., 1988*a*, 1988*b*) but not in others (Rofstad et al., 1988*a*; Ross et al., 1988; Steen et al., 1988; Rodrigues et al., 1988).

The fall in PCr usually seen in enlarging tumours has often been attributed to PCr being used to synthesise ATP in cells that were becoming hypoxic as a result of impaired tumour circulation (Evanochko et al., 1984; Remy et al., 1987; Rofstad et al., 1988*a*) and this is said to compensate for the 'inefficiency' of the glycolytic pathway as a source of ATP (Rofstad et al., 1988; Evanochko et al., 1984; Remy et al., 1987; Glickson et al., 1989).

$$\text{ADP} + \text{PCr} \rightleftharpoons \text{ATP} + \text{Cr}$$
creatine kinase

In practice, however, the amount of PCr in a tumour is far too small (typically 1–2 $\mu$mol g$^{-1}$ wet weight) to make a significant contribution to the ATP supply. Consider that tumour ATP turnover is probably about 6 $\mu$mol min$^{-1}$ g$^{-1}$: conversion of all the PCr would supply ATP for only 10–20 s, whereas the fall in PCr concentration actually takes place over a period of days, or even weeks.

What we observe in the $^{31}$P spectrum of a tumour is a slowly changing steady state in which the concentrations of the metabolites vary so as to maintain the maximum possible rate of ATP synthesis despite a steady fall in cellular oxygen concentration.

Do the $^{31}$P-NMR changes that we, and others, have observed in growing tumours reflect events in all the cells of a tumour? Or do they occur only in the necrotic cells, with the remainder being unaffected? This question has some practical importance: does $^{31}$P-NMR tell us about the proportion of the tumour that has become necrotic or does it tell us about the metabolism of viable but hypoxic cells?

We can answer these questions for tumours such as the GH3 prolactinoma and the NMU-induced mammary tumour in the present study, and also for the MOPC 104E mouse myeloma, described by Ng et al. (1982). In all these tumours the PCr signal is lost before there is a significant fall in the NTP peaks. This is clearly inconsistent with the hypothesis that the changes occur only in cells that have become necrotic, since necrotic cells have no NTP. If the tumour consisted of normal cells with both PCr and NTP and necrotic cells with neither PCr nor NTP one would observe a synchronous fall in the PCr and NTP signals as the necrotic fraction increased in the growing tumour. This was clearly not the case in the NMU-induced mammary tumour or the GH3 prolactinoma (see PCr/NTP ratios in Tables I and II), and in the larger NMU-induced mammary tumours PCr was undetectable despite strong NTP signals. Significant NTP resonances have been reported (Ross et al., 1988) even when there was 80–90% necrosis present in a C6 glioma, supporting the suggestion that the NMR signal comes from the viable cells.

We can conclude, for these tumours at least, that the $^{31}$P-NMR data tell us about the metabolic changes that occur in viable cells, and thus in cells that may be the target of therapy.

The authors would like to thank Dr S. Doak for histological preparation and assessment.

### References

ACKERMAN, J.J.H., GROVE, T.H., WONG, G.G., GADIAN, D. & RADDA, G.K., (1980). Mapping of metabolites in whole animals by 31P NMR using surface coils. *Nature*, **283**, 167.

ADAMS, D.A., DENARDO, G.L., DENARDO, S.J., CONBOY, C.B. & BRADBURY, E.M. (1985). $^{31}$P NMR analysis of metabolic status in KHJJ tumours. *Magn. Resonance Med.*, **2**, 419.

BERGMEYER, H.U. (ed.) (1974) *Methods of Enzymatic Analysis*, 2nd edn, p. 1777. Verlag Chemie: Weinheim.

CHANDRA RAJAN, J. & KLEIN, L. (1976). Determination of inorganic phosphorus in the presence of organic phosphorus and high concentrations of proteins. *Anal. Biochem.* **72**, 407.

CORBETT, R.J.T., NUNALLY, R.L., GIOVANELLA, B.C. & ANTICH, P.P. (1987). Characterization of the 31-P nuclear magnetic resonance spectrum from human melanoma tumors implanted in nude mice. *Cancer Res.*, **47**, 5065.

EVANOCHKO, W.T., NG, T.C. & GLICKSON, J.D. (1984). Application of *in vivo* NMR spectroscopy to cancer. *Magn. Resonance Med.*, **1**, 508.

FREEMAN, D., BARTLETT, S., RADDA, G. & ROSS, B.D. (1983). Energetics of sodium transport in the kidney. *Biochim. Biophys. Acta*, **762**, 325.

GADIAN, D.G. (1982) *Nuclear Magnetic Resonance and Its Applications to Living Systems*. Clarendon Press: Oxford.

GLICKSON, J.D., EVANOCHKO, W.T., SAKAI, T.T. & NG, T.C. (1989). *In vivo* NMR spectroscopy of tumors. In *NMR Spectroscopy of Cells and Organisms, Vol. 1*, Gupta R.K. (ed.). CRC Press: Boca Raton.

GRIFFITHS, J.R., BHUJWALLA, Z., COOMBES, R.C. & 10 others (1987). Monitoring cancer therapy by NMR spectroscopy. *Ann. NY, Acad. Sci.*, **508**, 183.

GRIFFITHS, J.R. & ILES, R.A. (1982). NMR studies of tumours. *Biosci. Rep.*, **2**, 719.

GRIFFITHS, J.R. & STEVENS, A.N. (1981). Topical magnetic resonance studies of tumours. *Biochem. Soc. Trans.*, **9**, 283P.

GRIFFITHS, J.R., STEVENS, A.N., ILES, R.A., GORDON, R.E. & SHAW, D. (1981). $^{31}$p-NMR investigation of solid tumours in the living rat. *Biosci. Rep.*, **1**, 319.

ILES, R.A., STEVENS, A.N., GRIFFITHS, J.R. & MORRIS, P.G. (1985). Phosphorylation of liver by 31P NMR spectroscopy, and its implications for metabolic control. *Biochem. J.* **229**, 141.

LOWRY, O.H. & LOPEZ, J.A. (1946). The determination of inorganic phosphate in the presence of labile phosphate esters. *J. Biol. Chem.*, **162**, 421.

MCGILVERY, R.W. & MURRAY, T.W. (1974). Calculated equilibria of phosphocreatine and adenosine phosphates during utilization of high energy phosphate by muscle. *J. Biol. Chem.*, **249**, 5845.

NG, T.C., EVANOCHKO, W.T., HIRAMOTO, R.N. & 6 others (1982). 31-P NMR spectroscopy of *in vivo* tumors. *J. Magn. Resonance*, **49**, 271.

PRICHARD, J.W., ALGER, J.R., BEHAR, K.L., DETROFF, O.A.C. & SHULMAN, R.G. (1983). Cerebral metabolic studies *in vivo* by $^{31}$P-NMR. *Proc. Natl Acad. Sci USA*, **80**, 2748.

REMY, C., ALBRAND, J.P., BENABID, A.L. & 4 others (1987). *In vivo* 31P NMR studies of $T_1$ and $T_2$ relaxation times in rat brain and rat brain tumours implanted in nude mice. *Magn. Resonance Med.*, **4**, 144.

RODRIGUES, L.M., MIDWOOD, C.J., COOMBES, R.C., STEVENS, A.N., STUBBS, M. & GRIFFITHS, J.R. (1988). 31-P Nuclear magnetic resonance spectroscopy studies of the response of rat mammary tumors to endocrine therapy. *Cancer Res.*, **48**, 89.

ROFSTAD, E.K., DE MUTH, P. & SUTHERLAND, R.M. (1988*a*). 31P NMR spectroscopy measurements of human ovarian carcinoma xenografts: relationship to tumour volume, growth rate necrotic fraction and differentiation status. *Radiother. Oncol.*, **12,** 315.

ROFSTAD, E.K., HOWELL, R.L., DEMUTH, P., CECKLER, T.L. & SUTHERLAND, R.M. (1988*b*) 31-P NMR spectroscopy *in vivo* of two murine tumor lines with widely different fractions of radiobiologically hypoxic cells. *Int. J. Radiat. Biol.*, **54,** 635.

ROSS, B.D., FREEMAN, D.M. & CHAN, L. (1984). Phosphorus metabolites by NMR. *Adv. Exp. Med. Biol.*, **178,** 455.

ROSS, B.D., HIGGINS, R.J., BOGGAN, J.E., KNITTEL, B. & GARWOOD, M. (1988). 31-P NMR spectroscopy of the in vivo metabolism of an intracerebral glioma in the rat. *Magn. Resonance Med.*, **6,** 403.

SHATTON, J.B., MORRIS, H.P. & WEINHOUSE, S. (1979). Creatine kinase activity and isozyme composition in normal tissues and neoplasms of rats and mice. *Cancer Res.*, **39,** 492.

SHUTTLEWOOD, R. & GRIFFITHS, J.R. (1982). The purine nucleotide profile in mouse, chicken and human dystrophic muscle: an abnormal ratio of inosine plus adenine nucleotides to guanine nucleotides. *Clin. Sci.*, **62,** 113.

STEEN, R.G., TAMARGO, R.J., MCGOVERN, K.A. & 4 others (1988). *In vivo* 31P NMR spectroscopy of subcutaneous 9L gliosarcoma: effects of tumour growth and treatment with 1,3-*bis* (2-chloroethyl)-1-nitrozurea on tumour bioenergetics and histology. *Cancer Res.*, **48,** 676.

STUBBS, M., RODRIGUES, L.M. & GRIFFITHS, J.R. (1988*b*). Comparison of 31-P spectra of animal tumours with histology. Second European Congress of NMR in Medicine and Biology, Berlin, p. 93.

STUBBS, M., RODRIGUES, L.M. & GRIFFITHS, J.R. (1988*c*). Correlation of $^{31}$P-NMR spectra with acid extracts and histology in a growth study of some animal tumours. *Proc. 7th Ann. Meeting Soc. Magn. Resonance Med.*, **1,** 407.

STUBBS, M., RODRIGUES, L.M. & GRIFFITHS, J.R. (1989). Potential artefacts from overlying tissues in $^{31}$-P NMR spectra of subcutaneously implanted rat tumours. *NMR in Biomed.*, **1,** 165.

STUBBS, M., VANSTAPEL, F., RODRIGUES, L.M. & GRIFFITHS, J.R. (1988*a*). Phosphate metabolites in rat skin *NMR Biomed.*, **1,** 50.

TOZER, G.M., BHUJWALLA, Z.M., GRIFFITHS, J.R. & MAXWELL, R.J. (1989). Phosphorus-31 magnetic resonance spectroscopy and blood perfusion of the RIF-1 tumor following X-irradiation *Int. J. Radiat. Oncol. Biol. Phys.*, **16,** 155.

VEECH, R.L., LAWSON, J.W.R., CORNELL, N.W. & KREBS, H.A. (1979). Cytosolic phosphorylation potential. *J. Biol. Chem.*, **254,** 6538.

WEHRLE, J.P., LI, S.J., RAJAN, S.S., STEEN, R.G. & GLICKSON, J.D. (1987). $^{31}$P and $^{1}$H NMR spectroscopy of tumours *in vivo*: untreated growth and response to chemotherapy. *Ann. NY. Acad. Sci.*, **508,** 200.

WILLIAMS, J.C., GUSTERSON, B., HUMPHREYS, J. & 4 others (1981). *N*-methyl-*N*-nitrosourea-induced rat mammary tumors. Hormone responsiveness but lack of spontaneous metastasis. *J. Natl Cancer Inst.*, **66,** 147.